# An eHealth intervention for patients with a low socioeconomic position during their waiting period preceding cardiac rehabilitation: a randomized feasibility study

**DOI:** 10.1093/ehjdh/ztae084

**Published:** 2024-11-14

**Authors:** Jasper S Faber, Jos J Kraal, Nienke ter Hoeve, Isra Al-Dhahir, Linda D Breeman, Niels H Chavannes, Andrea W M Evers, Hans B J Bussmann, Valentijn T Visch, Rita J G van den Berg-Emons

**Affiliations:** Department of Human-Centered Design, Faculty of Industrial Design Engineering, Delft University of Technology, Landbergstraat 15, Delft 2628 CE, The Netherlands; Department of Human-Centered Design, Faculty of Industrial Design Engineering, Delft University of Technology, Landbergstraat 15, Delft 2628 CE, The Netherlands; Department of Rehabilitation Medicine, Erasmus MC, Rotterdam, The Netherlands; Capri Cardiac Rehabilitation, Rotterdam, The Netherlands; Faculty of Social and Behavioural Sciences, Leiden University, Leiden, The Netherlands; Faculty of Social and Behavioural Sciences, Leiden University, Leiden, The Netherlands; Department of Public Health and Primary Care, Leiden University Medical Centre, Leiden, The Netherlands; National eHealth Living Lab, Leiden University Medical Centre, Leiden, The Netherlands; Department of Human-Centered Design, Faculty of Industrial Design Engineering, Delft University of Technology, Landbergstraat 15, Delft 2628 CE, The Netherlands; Faculty of Social and Behavioural Sciences, Leiden University, Leiden, The Netherlands; Medical Delta, Leiden University, Delft University of Technology, Erasmus University, Delft, The Netherlands; Department of Rehabilitation Medicine, Erasmus MC, Rotterdam, The Netherlands; Department of Human-Centered Design, Faculty of Industrial Design Engineering, Delft University of Technology, Landbergstraat 15, Delft 2628 CE, The Netherlands; Department of Rehabilitation Medicine, Erasmus MC, Rotterdam, The Netherlands; Capri Cardiac Rehabilitation, Rotterdam, The Netherlands

**Keywords:** Cardiac rehabilitation, Socioeconomic factors, Telemedicine, Time to treatment

## Abstract

**Aims:**

Cardiac rehabilitation (CR) shows lower effectiveness and higher dropouts among people with a low socioeconomic position (SEP) compared to those with a high SEP. This study evaluated an eHealth intervention aimed at supporting patients with a low SEP during their waiting period preceding CR.

**Methods and results:**

Participants with a low SEP in their waiting period before CR were randomized into an intervention group, receiving guidance videos, patient narratives, and practical tips, or into a control group. We evaluated adherence (usage metrics), acceptance (modified Usefulness, Satisfaction, and Ease of use questionnaire), and changes in feelings of certainty and guidance between the waiting period’s start and end. Semi-structured interviews provided complementary insights. The study involved 41 participants [median interquartile range (IQR) age 62 (14) years; 33 males], with 21 participants allocated to the intervention group, using the eHealth intervention for a median (IQR) duration of 16 (10) days, using it on a median (IQR) of 100% (25) of these days, and viewing 88% of the available messages. Key adherence themes were daily routine compatibility and curiosity. Acceptance rates were 86% for usability, 67% for satisfaction, and 43% for usefulness. No significant effects on certainty and guidance were observed, but qualitative data suggested that the intervention helped to inform and set expectations.

**Conclusion:**

The study found the eHealth intervention feasible for cardiac patients with a low SEP, with good adherence, usability, and satisfaction. However, it showed no effect on feelings of certainty and guidance. Through further optimization of its content, the intervention holds promise to improve emotional resilience during the waiting period.

**Registration:**

This trial is registered as follows: ‘Evaluation of a Preparatory eHealth Intervention to Support Cardiac Patients During Their Waiting Period (PReCARE)’ at ClinicalTrials.gov (NCT05698121, https://clinicaltrials.gov/study/NCT05698121).

## Graphical Abstract

**Graphical Abstract:**
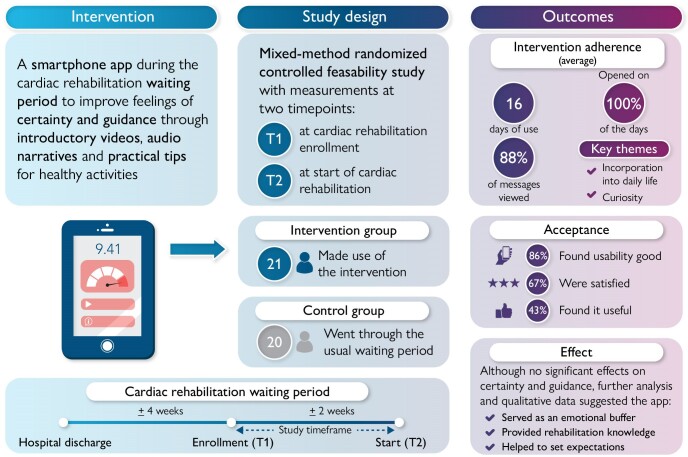

## Introduction

Cardiac rehabilitation (CR) is a multicomponent lifestyle intervention that includes information and coaching on healthy behaviour and supervised exercise training.^1,2^ Cardiac rehabilitation is crucial for cardiac patients to prevent secondary health problems and decrease mortality rates. It has been shown to improve patient outcomes like physical fitness and health-related quality of life.^3^ However, the effectiveness of CR is not uniformly experienced. Specifically, individuals with a low socioeconomic position (SEP) often show lower participation rates in these programmes and drop out more frequently.^4–6^ This disparity can be attributed to various barriers to participation,^7^ such as stressful life situations,^8^ environmental accessibility issues,^9^ inadequate social support,^10^ stigma and distrust in healthcare,^11^ and low health literacy.^12^ Due to these disparities, CR is not fully benefitting patients with a low SEP, underscoring the need for solutions to make CR more inclusive and accessible.

Our previous research highlights an opportunity to address the barriers faced by cardiac patients, especially those with a low SEP, during the waiting period between hospital discharge and the start of CR.^13^ This waiting period lasts, on average, 6 weeks.^14^ It is marked by emotional vulnerability and uncertainty, as patients often leave the hospital with unmet informational needs about their condition and self-care.^15–18^ The absence of adequate guidance during the waiting period, exacerbated by the initial shock of diagnosis or surgery, leads to a passive patient attitude^19–21^ and a disjointed transition between healthcare settings.^22^ Patients with a low SEP feel this lack of guidance more strongly. Their additional challenges increase their vulnerability and uncertainty during the waiting period.^23^ As a result, this group is less likely to adopt the necessary ‘readiness’ to successfully engage with CR. This leads to lower participation and higher dropout rates during CR.^24,25^

eHealth interventions are a promising strategy to overcome barriers that arise during the waiting period. They can better prepare cardiac patients with a low SEP for CR. For example, these interventions can fill the existing information and guidance gap by leveraging online information platforms^15^ and goal-monitoring tools.^26^ Due to rising healthcare costs, addressing these needs through face-to-face sessions during the waiting period may not be feasible.^27–29^ In theory, eHealth interventions offer a cost-effective alternative to face-to-face sessions.^28–32^ However, in practice, people with a low SEP often do not adhere to these interventions due to low technology access, low digital literacy, and other life priorities.^33,34^ The success of these interventions depends on tailoring them to the specific needs, abilities, and preferences of this group.^35^

We recently developed the Inclusive eHealth Guide (IeG) to support the design of tailored eHealth interventions according to the specific needs of individuals with a low SEP.^36^ The guide combines existing knowledge on barriers and facilitators in eHealth development for individuals with a low SEP.^37^ It considers, among others, the target group’s context, needs, preferences, and skills.^38^ We applied the IeG in a participatory design process of an eHealth intervention specifically for and with cardiac patients with a low SEP. The intervention addresses their needs during the waiting period before CR.^13^

This study aimed to evaluate the feasibility of this eHealth intervention tailored towards CR patients with a low SEP in the domains of adherence and acceptance. Additionally, it explored the effects of the eHealth intervention on feelings of certainty and guidance, factors associated with changes in these constructs, and dropout rates during subsequent CR.

## Methods

### Study design

The feasibility study was executed between February 2023 and September 2023 at Capri Cardiac Rehabilitation, a CR centre with sites in Rotterdam and The Hague (The Netherlands). The participants were randomized to an intervention group and control group, and outcomes were assessed at the start and end of the waiting period before CR started.

### Recruitment

Eligible participants were adults aged 18 or above living in a low SEP neighbourhood, referred to CR by their cardiologist, able to understand Dutch (with assistance), and had a mobile device with internet access. Postal codes of potential participants were sent to the principal investigator (J.S.F.) to assess neighbourhood SEP, based on the neighbourhood residents’ average income and education levels. We used a list of 40 neighbourhoods identified by the Dutch government for their socioeconomic challenges as a benchmark.^39^ A representative from the CR centre first contacted potential participants for consent. Interested patients were then contacted by the investigator (J.S.F.), who explained the study. If they agreed to participate, they received an information letter and had an appointment scheduled for the initial assessment.

### Measures

#### Adherence

Adherence to the intervention was measured using the metrics (i) *use period length*: the number of days between the first and last day the intervention was used; (ii) *percentage of active days*: percentage of days the intervention was used; (iii) *daily use time*: average time spent on the intervention per active day within the use period; and (iv) the *total number of viewed messages*.

#### Acceptance

Acceptance was measured using a modified Usefulness, Satisfaction, and Ease of use (USE) questionnaire. The original USE questionnaire consists of 30 items on a 7-point Likert scale focusing on usefulness, satisfaction, ease of use, and ease of learning.^40^ In alignment with the specific needs and challenges faced by our target population of individuals with a low SEP, we recognized that lengthy questionnaires often lead to disengagement among this group.^41^ Therefore, we adapted the original questionnaire to a more manageable version with only nine of the original items (three per category) on a 5-point Likert scale, focusing on usefulness, satisfaction, and ease of use (see Supplementary material online, *Appendix S1*). The questions retained were chosen for their relevance to the unique context and goals of the current intervention.

#### Certainty and guidance and influencing factors

We developed the Certainty and Guidance Questionnaire (CGQ), consisting of seven items measured using a 5-point Likert scale, for use in this study (see Supplementary material online, *Appendix S1*). High scores indicate good certainty and guidance. The questionnaire focuses on patient needs identified in a previous study.^13^ These needs include feeling certain during the waiting period, confidence to be physically active, managing expectations about the contents of CR, good management of emotions, the feeling of hope for future recovery, understanding the current health status, and feeling guided before the start of CR. The questions are derived from existing scales, including the Motivation for Traumatic Brain Injury Rehabilitation Questionnaire for motivation,^42^ the Patient Evaluation of Emotional Comfort Experienced for experienced emotional comfort,^43^ and the Credibility and Expectancy Questionnaire for expectancy and credibility^44^ to strengthen the validity of our measurements. Finally, to better understand the factors influencing changes in feelings of certainty and guidance, we explored associations between age, education level, length of waiting period, baseline level of CGQ, and the change in CGQ scores in both the intervention and control groups.

#### Qualitative insights

In line with our mixed methods approach, we complemented the quantitative data for adherence, acceptance, and feelings of certainty and guidance with qualitative insights collected with semi-structured interviews. We asked questions relating to reasons for adherence (e.g. Why did or did you not succeed in using the intervention daily?), acceptance (e.g. What was your experience with using the intervention?), and effects on feelings of certainty and guidance (e.g. How has the intervention been able to help you the most during the waiting period?) (see Supplementary material online, *Appendix S2*, for the full interview guide).

### Procedures

**Figure 1:**
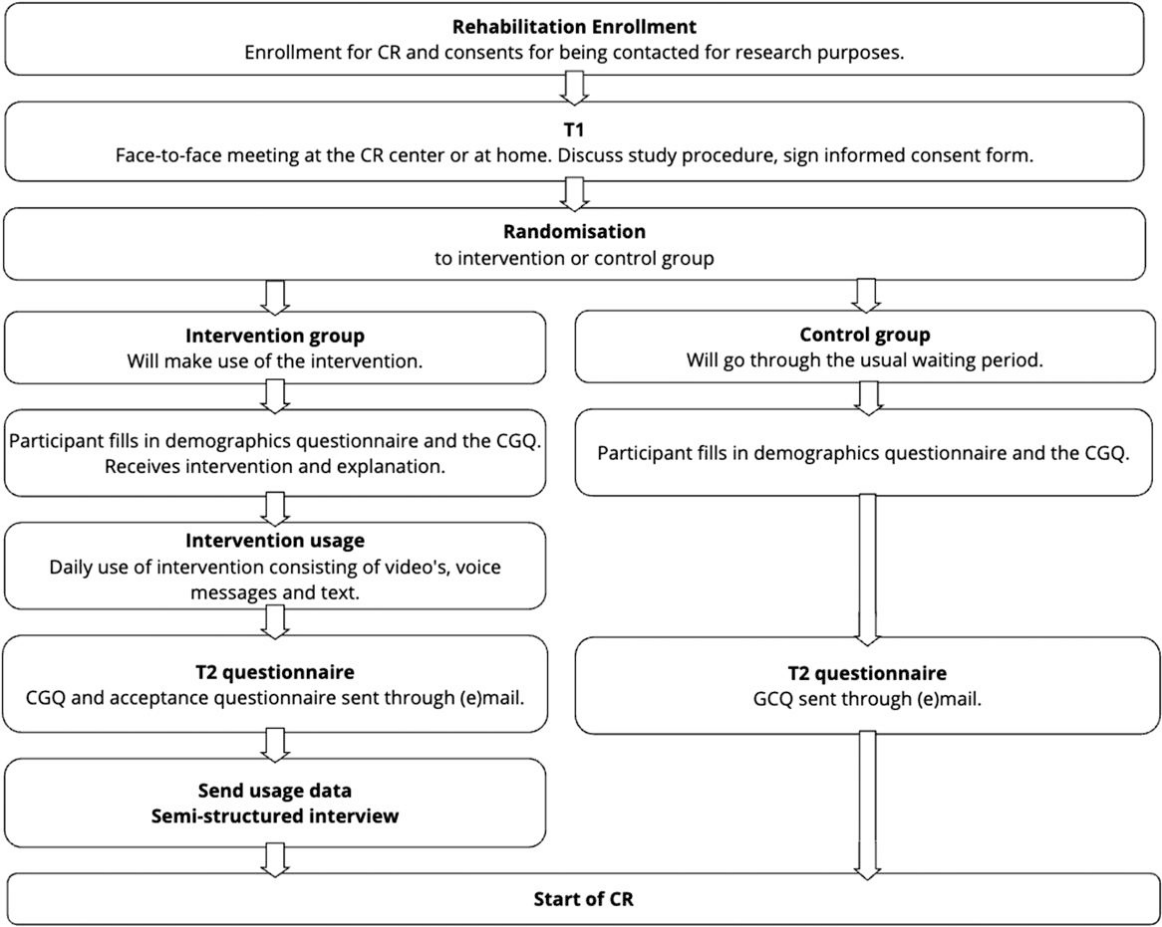
A visual overview of patient enrolment and study procedures. CR, cardiac rehabilitation; CGQ, Certainty and Guidance Questionnaire.

We performed assessments in both study groups during two contact moments: initially upon enrolment in CR (T_1_) and again just before the CR programme began, usually 2–8 weeks after T_1_ (T_2_) (see *Figure 1*). At T_1_, participants were briefed on the study, signed consent forms, and completed demographic and CGQ questionnaires. The intervention group received additional information about the smartphone app and help installing it. At T_2_, both groups completed a second CGQ questionnaire, with the intervention group also submitting usage data, filling out an acceptance questionnaire, and participating in a phone interview. Participants received a 20-euro gift voucher for their participation.

### Intervention

The CapriXpress application is a tailored digital intervention developed to support patients with a low SEP during their waiting period between discharge from the hospital and the start of their CR. This intervention was co-designed in a participatory design study with the target group.^13^ The CapriXpress application addresses the need for certainty and guidance for people with a low SEP during their waiting period. To enhance patient adherence and acceptance of the intervention, we ensured that the intervention is grounded in established theoretical frameworks, namely the taxonomy of behaviour change techniques (BCTs)^45^ and the persuasive systems design (PSD) model^46^ (see *Table 1*). Combining the two frameworks is valuable as it combines the rigour of scientifically validated methods for behaviour change with engaging, user-focused aspects of persuasive technology design.^47^ Additionally, the intervention integrates recommendations derived from our previously developed IeG,^36^ which served as a foundational resource (see *Table 1*).

**Table 1:** Intervention features that address adherence and acceptance, linked to principles from the Inclusive eHealth Guide, behaviour change technique, and persuasive systems design framework, and features that address patient needs as identified in our prior study

Features addressing adherence and acceptance				
Number	Feature	IeG recommendation	BCT	PSD principle
1.1	Limited number of daily messages	Realistic, achievable goals, align with life situation	Graded tasks	Reduction
1.2	Playful interface	Positive approach		Liking
1.3	Simple interface	Simplicity		Reduction
1.4	Done-pile tracker	Short-term goals, apply gamification	Self-monitoring/feedback	Self-monitoring
1.5	Bag upgrade	Reward for adherence, apply gamification	Non-specific reward	Rewards
1.6	Use of multimedia and simple language	Simplify communication		Tailoring
1.7	Tone of voice	Positive approach		
1.8	Notification	Send reminders		Reminders
1.9	Support page	Offer technical support		

Features addressing patient needs		
Number	Feature	Needs
2.1	Calendar-based progression	Certainty during waiting period, pre-CR guidance
2.2	Information provided by healthcare provider	Certainty during waiting period, pre-CR guidance
2.3	Video introductions	Certainty during waiting period, CR expectancy
2.4	Spoken peer stories	Certainty during waiting period, managing emotions, future recovery
2.5	Textual advice	Physical activity confidence, health status understanding

Inclusive eHealth Guide (IeG)^36^, behaviour change technique (BCT) taxonomy^48^, and persuasive system design (PSD) model,^46^ and features that address patient needs as identified in our prior study.

**Figure 2:**
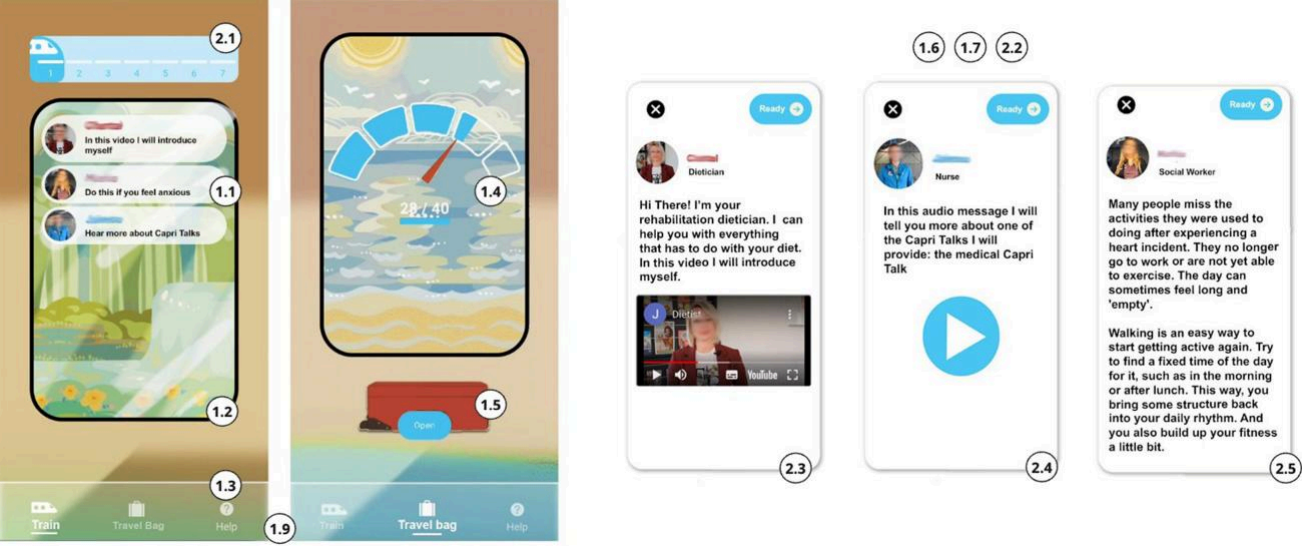
Key interface screens from the CapriXpress intervention. From left to right: journey-based progression home page, done-pile tracker and travel bag, and multimedia messages from healthcare professionals.

The content of the CapriXpress application is divided into concise, manageable units, with a limited number of messages presented per day (*Figure 2*, 1.1). The interface is designed to be playful, aesthetically pleasing, and simple to understand (*Figure 2*, 1.2 and 1.3). A ‘travel bag’ feature stores completed messages, giving patients a sense of achievement. When the bag is filled to a certain level, it receives an aesthetic upgrade as a reward (*Figure 2*, 1.4 and 1.5). The information is primarily conveyed through multimedia formats and is articulated in easily understandable language, adopting a positive tone (*Figure 2*, 1.6). A push notification is sent if the participant has not engaged with the intervention for two consecutive days. The ‘help’ section provides contact information for research or application-related questions (*Figure 2*, 1.9).

Several intervention features have been implemented to address the target group’s needs during the waiting period. The application uses a calendar-based train journey metaphor to symbolize the patient’s progression towards the start of CR (*Figure 2*, 2.1). This progression occurs automatically over time, aiming to provide a sense of certainty during the waiting period. The app delivers a total of 43 multimedia messages, defaulting to three per day, with the frequency adjusting based on the patient’s specified CR start date. Patients can choose from three message types: introductory videos from healthcare providers like a physiotherapist, dietitian, and psychologist to inform and connect with the CR team (*Figure 2*, 2.3); audio narratives from former patients to offer emotional support and hope (*Figure 2*, 2.4); and actionable advice promoting healthy activities and improving understanding of their condition and the rehabilitation process (*Figure 2*, 2.5).

### Data analysis

We analysed our quantitative data in RStudio (Version 2023.06.0, Posit Software, PBC). We utilized medians, interquartile ranges (IQRs), and non-parametric statistical tests to ensure robustness and minimize assumptions about data distribution, given our limited sample size. The level of significance was set at *P* ≤ 0.05.

We transformed the raw intervention usage data into specific metrics to evaluate adherence. We calculated the use period length from the first day of use (T_1_) to the last completed message. The percentage of active days was determined by dividing the number of days the intervention was used by the total use period length. Daily use time was derived by summing the duration of all visits and dividing it by the number of active days. We also totalled the number of viewed messages. Adherence was considered satisfactory if participants used the intervention on at least half the days and viewed more than half the available messages.

To analyse the acceptance from the adapted USE questionnaire, we classified the Likert scores as negative (1 or 2), neutral (3), or positive (4 or 5) and calculated the percentages of participants in each category. We then calculated individual scores for usability, usefulness, and satisfaction and determined the median, IQR, minimum, and maximum scores for these metrics across intervention group participants. Overall acceptance was similarly assessed using these statistics. For this prototype, a score was deemed good if over 60% of the participants rated it positively.

To assess the intervention’s effect on certainty and guidance during the waiting period, we calculated the median Likert scores for the CGQ items for each participant and determined group medians for both the intervention and control groups. Wilcoxon rank-sum and Mann–Whitney *U* tests were used to assess within- and between-group differences, respectively. Rank correlation tests examined the relationship between changes in CGQ scores and factors such as age, education level, initial CGQ scores, and waiting period length. Fisher’s exact test was used to analyse differences in dropout rates.

For the qualitative data, we performed a thematic analysis^49^ using ATLAS.ti (Version 9.1.3, ATLAS.ti Scientific Software Development GmbH). Interviews were transcribed verbatim, followed by coding individual quotations and corresponding interpretations. These codes were then grouped into overarching themes related to the outcome measures, such as adherence, acceptance, and impact on feelings of certainty and guidance.

### Ethics and data management

This study adhered to the Declaration of Helsinki principles and was approved by the Medical Ethics Committee of Erasmus MC (MEC-2022-0483) and registered in clinicaltrials.gov (NCT05698121). Written informed consent was obtained from all study participants.

## Results

### Participants

**Figure 3:**
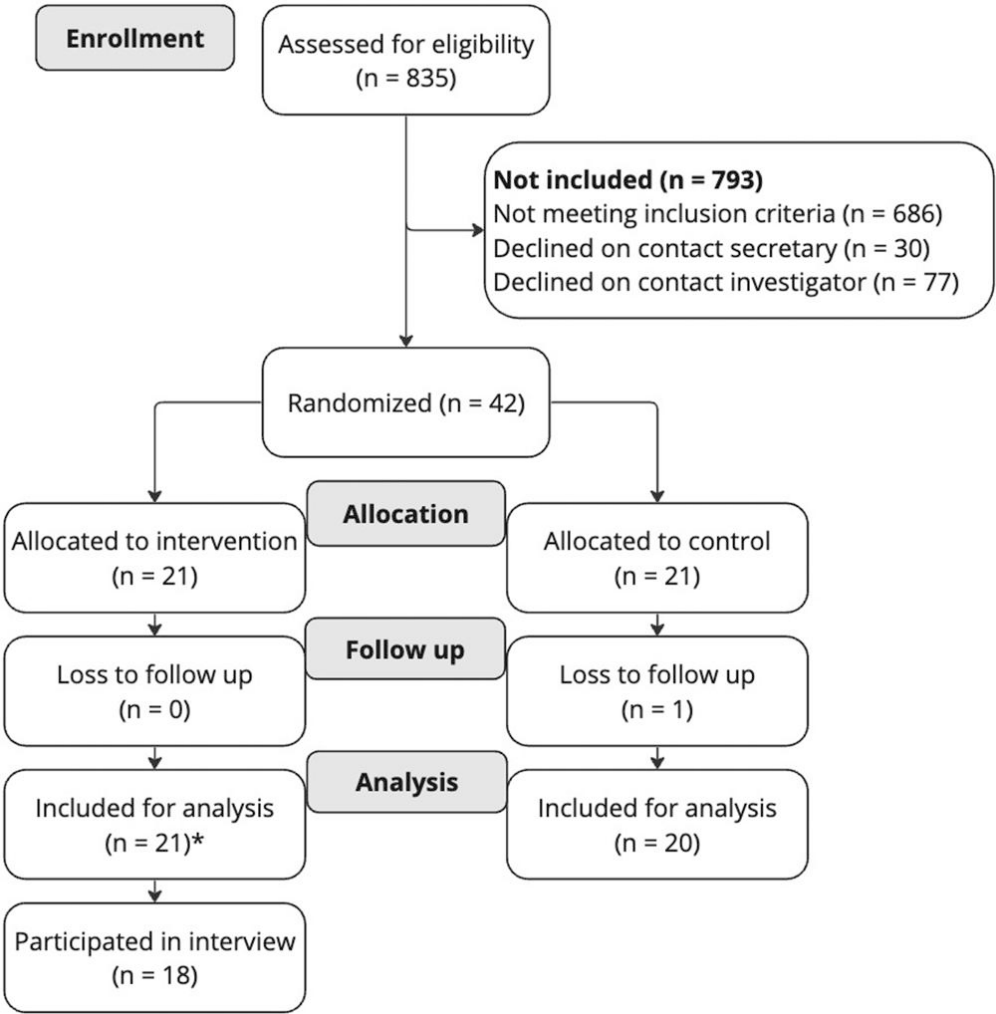
Flowchart participant inclusion. Asterisk denotes 19 participants were included for the intervention adherence analysis.

Out of the 835 patients referred to the CR centre during the recruitment period (January 2023 to June 2023), 149 patients (18%) were eligible, of which 42 patients (28%) consented to participate. Frequently reported reasons for non-participation were personal circumstances, logistical issues, lack of interest, technological barriers, and language and cognitive barriers. Twenty-one participants were assigned to the intervention group and 21 to the control group (see *Figure 3*). One participant in the control group dropped out during the study due to the burden of participation. Eighteen participants from the intervention group participated in a semi-structured interview, and 19 participants from the intervention group sent their usage data for the adherence analysis. The majority of the sample was male (80%), with a median (IQR) age of 62 (14) years. Ischaemic heart disease was the most common condition (63%). The median (IQR) waiting time from hospital discharge to the start of CR was 55 (43) days and 29 (13) days from enrolment at the CR facility to the beginning of the programme (see *Table 2* for more details).

**Table 2:** Participant characteristics

Characteristic	Intervention group (*n* = 21)	Control group (*n* = 20)	Sample (*n* = 41)
Demographics			
Male, *n* (%)	17 (81)	16 (80)	33 (80)
Age (years), median (IQR)	63 (13)	59 (13)	62 (14)
Low education, *n* (%)	15 (71)	18 (90)	33 (80)
Employed, *n* (%)	4 (19)	6 (30)	10 (25)
Unemployed, *n* (%)	1 (5)	2 (10)	3 (7)
Retired, *n* (%)	11 (52)	7 (35)	18 (44)
Unfit for work, *n* (%)	5 (24)	5 (25)	10 (24)
Medical history, *n* (%)			
Ischaemic heart disease	15 (70)	11 (55)	26 (63)
Cardiac arrhythmia	2 (10)	1 (5)	3 (7)
Other, cardiac disease	4 (20)	7 (35)	11 (27)
Other, non-cardiac disease	0 (0)	1 (5)	1 (3)
Waiting time, days, median (IQR)			
Hospital discharge—start CR	66 (31)	43 (32)	55 (43)
Enrolment CR—start CR	28 (13)	29 (11)	29 (13)

### Adherence to the intervention

**Figure 4:**
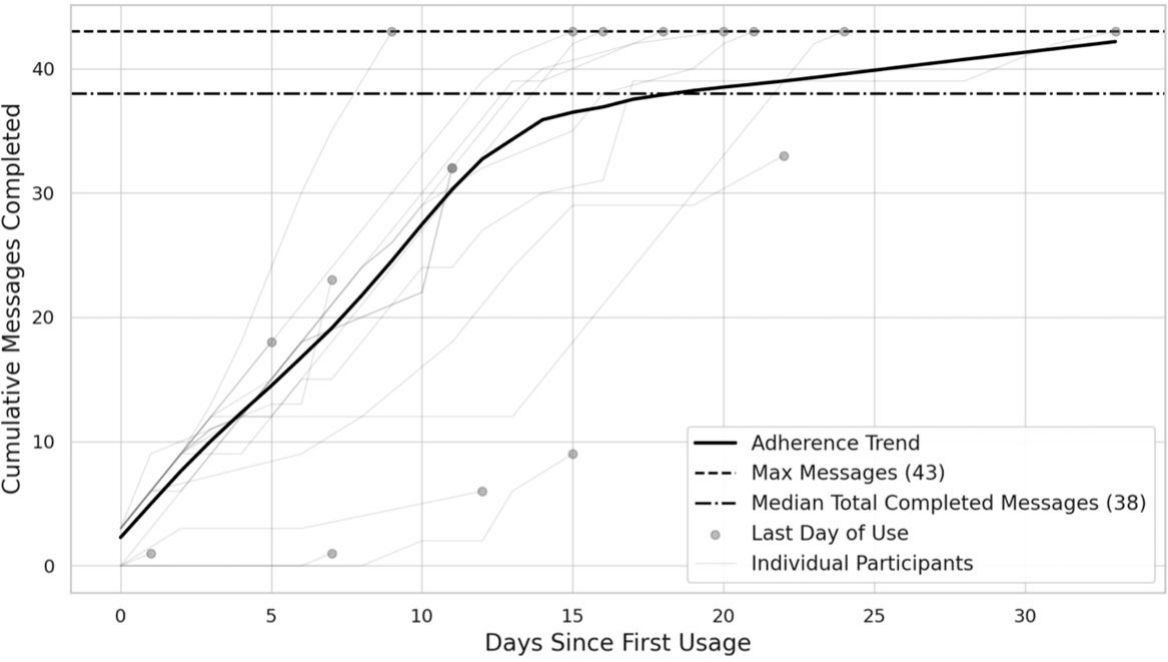
Cumulative messages completed vs. days since the first usage (median ± interquartile range).

The median (IQR) *use period length* was 16 (10) days. During this period, the median (IQR) percentage of the days the participants accessed the application was 100% (25), with a median (IQR) *daily use time* of 4 (2) min. Sixty-seven per cent of the participants opened the application every day. Regarding content interaction, the median (IQR) *total number of messages viewed* was 38 (24) out of 43. Half of the participants viewed all the messages available. *Figure 4* presents the relationship between the number of days since first usage and the cumulative messages completed by participants. The trend line shows continuous completion of messages over time with a slight decrease in the number of messages completed each day after ∼2 weeks. Two qualitative themes related to these adherence patterns emerged (see Supplementary material online, *Appendix S3*, for a complete overview of the qualitative themes). First, almost three-quarters of the participants stated that the intervention aligned well with their daily routines. As one participant expressed:

We used to sit in the morning for coffee. Yeah, we would sit down for a while, and I received them [the messages], and then I had my phone in my hand. Well, I went through it; I even turned it on so that the lady could listen along and that way. Yeah, it's also at a fixed time. You have to be careful not to leave it for a whole week and then review it after a week. Because that will not work, I think. If you throw everything together, it is just a matter of sifting through it and fulfilling a duty. [Male, 73]

Second, we found that more than half of the interviewed participants cited curiosity as their driving factor for usage. As one participant stated:

I was curious about it every day. I also opened it every day. I went through the entire program. I was, well, actually, looking forward to seeing what news they had to say today. Yeah, it was actually more curiosity. [Male, 69]

### Acceptance of the intervention

Seventy-one per cent of the participants displayed overall positive acceptance. We found that 86% of the participants were positive about the intervention’s usability, and 67% were satisfied. Forty-three per cent felt that the intervention was useful for them (see *Table 3* for a complete overview of the acceptance scores). Within the qualitative data, we found that participants mainly appreciated the *ease of use* and the *playful interface*. As one participant expressed:

**Table 3:** Overview of acceptance scores displayed as median interquartile ranges and percentages of participants in the categories positive, neutral, and negative on usability, satisfaction, and usefulness

Question	Median (IQR), minimum–maximum	Positive (4–5) *n* (%)	Neutral (3) *n* (%)	Negative (1–2) *n* (%)
Overall	3.8 (0.8), 2.7–5.0	15 (71)	6 (29)	0 (0)
Usability	4.0 (1.0), 2.7–5.0	18 (86)	3 (14)	0 (0)
Is easy to use	4 (1), 3–5	18 (86)	3 (14)	0 (0)
Required no effort	4 (1), 2–5	19 (90)	1 (5)	1 (5)
Allowed to perform well	4 (2), 2–5	15 (71)	5 (24)	1 (5)
Satisfaction	4.0 (0.7), 2.3–5.0	14 (67)	6 (28)	1 (5)
Is fun to use	4 (1), 3–5	14 (67)	7 (33)	0 (0)
Would recommend to others	4 (1), 2–5	18 (85)	2 (10)	1 (5)
Aligns with needs	4 (1), 1–5	14 (67)	3 (14)	4 (19)
Usefulness	3.3 (1.0), 2.3–5.0	9 (43)	10 (47)	2 (10)
Is useful	4 (1), 3–5	13 (62)	8 (38)	0 (0)
Aligns with needs	3 (1), 2–5	7 (33)	11 (52)	3 (15)
Aligns with expectations	4 (1), 2–5	11 (52)	6 (29)	4 (19)

Well, you know, I found it enjoyable. It’s more enjoyable than just a boring list or something, you know. Yeah, it’s funny that they thought of it like, oh yeah, let’s pretend it’s a journey. With your stories in a suitcase, very amusing. You’re on a journey to your rehabilitation. [Female, 60]

We also found qualitative themes that related to the usefulness. More than half of the participants suggested the need for more *personally relevant information* better aligned with their health concerns and the severity of their conditions. As one participant expressed:

All those social workers and such…For me, I think it's not interesting. I only do it to become physically well. That's my goal. I don't think I have any other issues. I think the app is limited in that aspect. [Male, 76]

Additionally, approximately half the participants suggested a need for *additional depth and detail* in the provided information. As one participant expressed:

The dietitian gave a very brief explanation of what she does. […] But she didn’t really delve into the topic. For example, what can you tell about your sugar or salt levels being too high? What are the consequences of that? Could you get paralysis? Could you have a heart attack? So, the information was lacking, in my opinion. [Male, 63]

### Effects on certainty and guidance

We found no significant changes in CGQ scores between T_1_ and T_2_ in both the intervention [Δ = −0.14 (IQR 0.57), *P* = 0.94] and control group [Δ = −0.07 (IQR 0.32), *P* = 0.51]. In addition, we did not find a significant difference in the changes in CGQ scores between the two groups (*P* = 0.51). The qualitative data highlighted areas that did address improvements in feelings of certainty and guidance during the waiting period. Participants suggested that the intervention improved their expectancy about the CR, as one participant mentioned:

You know, when all those people introduced themselves and told stories about different participants. Yeah, that was nice because you know beforehand what to expect when you start the rehabilitation. So, that was quite pleasant. [Male, 73]

In addition, it helped them to feel generally better informed about their current condition by providing additional knowledge they usually would not receive. As one participant expressed:

I received a lot of information that I wouldn’t normally get. If you haven’t had a heart attack, you don’t even think about all the information you’ve received. So, for me, it was a kind of recognition. And it was very good. So, as I said, it made me wise. [Male, 63]

Finally, the participants highlighted the intervention helped to reduce uncertainties by providing guidance during the waiting period gap. As expressed by one participant:

Well, in terms of reducing uncertainties, the app did help me because if you didn’t have that app, you would fall into a void between being discharged from the hospital and starting rehabilitation. So, in that sense, the app was able to provide assistance in filling that void at some point. [Male, 74]

### Factors associated with the effect on certainty and guidance

The length of the waiting period had a significant negative correlation with the change in CGQ score in the control group (*ρ* = −0.51, *P* = 0.02) but not in the intervention group (*ρ* = −0.04, *P* = 0.86). In addition, higher CGQ scores at T_1_ were negatively correlated with changes in CGQ scores in both the intervention group (*ρ* = −0.56, *P* = 0.01) and the control group (*ρ* = −0.49, *P* = 0.03). Age and education were not significantly correlated with changes in CGQ scores in both intervention (*P* = 0.16 and *P* = 0.26, respectively) and control (*P* = 0.94 and *P* = 0.66, respectively) groups.

### Effects on dropout during CR

Two (10%) of the participants dropped out of the subsequent CR programme in the control group compared to none in the intervention group. This difference was, however, not significant (*P* = 0.23).

## Discussion

### Principal findings

In this study, we evaluated the feasibility and explored the effects on feelings of certainty and guidance and on dropouts of a newly developed eHealth intervention for CR patients with a low SEP during their waiting period before starting a CR programme. We found good adherence with the participants often using the intervention daily and engaging with 88% of the messages. Most participants (71%) displayed positive overall acceptance of the intervention. However, only 43% were positive about usefulness. The intervention did not affect feelings of certainty and guidance (CGQ) or dropout rate. However, while the length of the waiting period was negatively associated with feelings of certainty and guidance in the control group, no such association was observed in the intervention group. Qualitative feedback suggested that the intervention had helped participants to set expectations and be better informed about their condition and CR journey.

Usage data indicated consistent adherence over time, although there was a slight reduction in daily message interactions after the first 2 weeks. This decrease aligns with the intervention’s dynamic content distribution system, which recalibrates the frequency of messages once participants enter their CR start dates. When the starting date is further in the future, the system automatically reduces the number of messages provided daily to extend the usage period. Despite this, continued message views suggest sustained adherence, which contrasts with the relatively low eHealth adherence often observed in people with a low SEP.^33,34^ Many participants cited the intervention’s integration into daily routines as crucial. Past studies indicate that individuals with a low SEP often face stressful daily challenges, limiting their time and cognitive capacity for engaging with eHealth interventions.^50^

Additionally, curiosity was reported as a key factor in the patient’s adherence to the intervention, aligning with the gamification theory that presents curiosity as a strategy to enhance engagement with a system.^51^ This facet of the intervention might have been an important contributor to the observed adherence. Regarding acceptance, the intervention’s well-received usability contrasts with findings in existing literature. Typically, individuals with a low SEP encounter more challenges with the usability of eHealth interventions.^52–55^ The intervention’s consistent adherence and positive overall acceptance could be attributed to its participatory design, which followed the IeG’s recommendations for equitable eHealth development. Failing to achieve adherence and acceptance could negatively influence overall effectiveness, irrespective of any inherent benefits of the intervention.^56^ Given our promising outcomes on adherence and acceptance, we recommend future researchers to apply the IeG and engage in tailored participatory approaches to develop eHealth interventions for individuals with a low SEP in different settings.

While we did not find a significant intervention effect in this feasibility study on feelings of certainty and guidance or dropout in subsequent CR, we did find some trends pointing towards potential intervention effects. First, the qualitative findings suggest that the participants felt that the intervention contributed to their feelings of certainty and guidance. The interview results suggested improved expectations for future CR, better information, and guidance during the waiting period. These insights hint at the intervention enhancing participant readiness and motivation for CR. Second, the finding that the length of the waiting period was negatively associated with the change in feelings of certainty and guidance in the control group but not in the intervention group suggests that the intervention could serve as an emotional buffer for patients facing longer waiting periods. Qualitative feedback further supports this, with participants reporting that the intervention helped to set expectations and provide information regarding their rehabilitation journey. Although it did not directly improve certainty and guidance, the intervention might have fostered a sense of readiness for rehabilitation by giving information and early engagement with the programme. In future versions of the intervention, its content should be focused more directly on improving the patient’s feeling of certainty and guidance. Lastly, although the difference in dropout rates between the intervention and control group was not significant, the 10% dropout rate in the control group is consistent with the general dropout rate in CR.^57^ The absence of dropouts in the intervention group could suggest that the intervention may have boosted participant’s commitment to CR. This should, however, be confirmed in a sufficiently powered trial.

Our study found participants preferred more personally relevant content and additional depth and detail in information. This suggests that the intervention’s one-size-fits-all approach may not meet varying needs for content depth and relevance. This desire for personalized content also aligns with previous research findings.^58,59^ Personalized information, as opposed to generic information, has demonstrated a greater positive impact on well-being,^60^ health plan decision-making,^61^ and lifestyle behaviour.^62^ Within CR, several studies have shown to be effective that employed dynamic personalization techniques, such as using initial screenings^63^ or artificial intelligence algorithms to adapt the content and delivery in real-time based on user’s interactions and responses.^60,64^ Future research could explore developing personas or patient profiles reflecting diverse content needs based on health concerns, condition severity, and motivation.^65^ These profiles would guide the creation of tailored pathways for pre-CR content, accommodating different patient types during their waiting period. Pathways may vary by exercise difficulty aligned with disease severity and information delivery adjusted to individual knowledge and health literacy levels.

### Strengths and limitations

This feasibility study lays the groundwork for designing effective interventions for patients with a low SEP during their waiting period before starting CR. Such studies are essential for refining intervention designs to improve impact when scaled up.^66^ A strength of our research is the mixed methods approach, which offered insightful explanations for our findings and laid a foundation for future research and development. Additionally, it is noteworthy that we have maintained a participant retention rate similar to other trials conducted at Capri Cardiac Rehabilitation,^67,68^ despite our emphasis on people with a low SEP, who are typically underrepresented in these earlier trials.

While our study provides important exploratory insights, the results should be approached with caution and seen as suggestive rather than conclusive. It is important to consider the small sample size and single-centre data collection when interpreting these findings, as the limited sample size particularly affects the robustness of the *P*-values. While our use of a self-designed questionnaire may have affected validity, we mitigated this by basing our questions on established instruments and employing a mixed methods approach to triangulate the quantitative data with qualitative data.^69^ Additionally, excluding a participant who dropped out from the analysis could further limit our study’s integrity. We recommend validating our results in a larger, more robust trial, with validated instruments, conducted across multiple rehabilitation centres.

Another potential limitation of our study lies in the composition of our participant sample, which, due to neighbourhood-level sampling, possibly included individuals with higher SEP. Although our data indicate a low percentage of highly educated individuals within our sample, this metric alone may not provide a comprehensive picture of SEP. Socioeconomic position is a multi-faceted concept influenced by various factors beyond formal education levels. Additionally, our recruitment approach might have favoured those more experienced with digital tools and comfortable in their current situation. Additionally, the interpretability of our results may have been affected by technical difficulties encountered during the early phase of the study, resulting in some participants not receiving messages for a few days. While this issue was promptly resolved, it might have influenced acceptance and adherence scores. As we plan for a larger, more robust trial, it is crucial to thoroughly test the intervention’s technical functionality before its commencement.

Finally, a notable limitation of our study concerns the interpretability of the CGQ scores, particularly due to the timeframe of the intervention and its overlap with interactions (e.g. scheduling appointments and intake sessions) in both the intervention and control groups at the CR facility. Most of these facility interactions occur in the final weeks, coinciding with the period when we evaluated the intervention’s effect. Moreover, the duration of intervention use, approximately 2 weeks, was relatively brief when contrasted with the average waiting period of 8 weeks in the Capri Cardiac Rehabilitation centre. These limitations might explain the discrepancy between the quantitative and qualitative findings. For future research, initiating the intervention immediately at hospital discharge would be beneficial, thereby exposing patients during the entire waiting period. The final measurements could be conducted before interactions with healthcare providers at the CR centre to minimize their influence on feelings of certainty and guidance.

## Conclusions

The developed eHealth intervention was well adhered to and accepted by the target group. Yet, usefulness should be improved, and we did not find effects on feelings of certainty and guidance or dropouts. Despite this, the findings from this feasibility study yield important insights into the design of eHealth interventions tailored to people with a low SEP. Through further optimization, for example through personalization and an extended timeframe for offering the intervention, the intervention holds promise as an effective tool to enhance participation in CR and improve adherence among patients with a low SEP, thereby mitigating health disparities in CR and improving its effectiveness. While researchers should acknowledge the limitations of this feasibility study, including its small sample size and focus on a single centre, it represents a first step towards equitable eHealth interventions. Healthcare professionals and intervention developers can leverage these findings to develop and tailor interventions that align with the needs and preferences of individuals with a low SEP, thereby improving their adherence and acceptance.

## Supplementary Material

ztae084_Supplementary_Data

## Data Availability

Data available on request.

## References

[1] Piepoli MF, Hoes AW, Agewall S, Albus C, Brotons C, Catapano AL, et al 2016 European guidelines on cardiovascular disease prevention in clinical practice: the sixth joint task force of the European Society of Cardiology and other societies on cardiovascular disease prevention in clinical practice (constituted by representatives of 10 societies and by invited experts)Developed with the special contribution of the European Association for Cardiovascular Prevention & Rehabilitation (EACPR). Eur Heart J 2016;37:2315–2381. 27222591 10.1093/eurheartj/ehw106 PMC4986030

[2] Revalidatiecommissie Nederlandse Vereniging Voor Cardiologie & Nederlandse Hartstichting . Multidisciplinaire richtlijn hartrevalidatie. *Nederlandse Vereniging Voor Cardiologie*. 2011.

[3] Eijsvogels TMH, Maessen MFH, Bakker EA, Meindersma EP, van Gorp N, Pijnenburg N, et al Association of cardiac rehabilitation with all-cause mortality among patients with cardiovascular disease in The Netherlands. JAMA Netw Open 2020;3:e2011686. 32716516 10.1001/jamanetworkopen.2020.11686 PMC12124693

[4] Valencia HE, Savage PD, Ades PA. Cardiac rehabilitation participation in underserved populations. Minorities, low socioeconomic, and rural residents. J Cardiopulm Rehabil Prev 2011;31:203–210. 21705915 10.1097/HCR.0b013e318220a7da

[5] Harlan WR III, Sandler SA, Lee KL, Lam LC, Mark DB. Importance of baseline functional and socioeconomic factors for participation in cardiac rehabilitation. Am J Cardiol 1995;76:36–39. 7793400 10.1016/s0002-9149(99)80797-8

[6] Ades PA, Khadanga S, Savage PD, Gaalema DE. Enhancing participation in cardiac rehabilitation: focus on underserved populations. Prog Cardiovasc Dis 2022;70:102–110. 35108567 10.1016/j.pcad.2022.01.003 PMC9119375

[7] Shanmugasegaram S, Oh P, Reid RD, McCumber T, Grace SL. Cardiac rehabilitation barriers by rurality and socioeconomic status: a cross-sectional study. Int J Equity Health 2013;12:72–72. 23985017 10.1186/1475-9276-12-72 PMC3765803

[8] Marmot M . Social determinants of health inequalities. Lancet 2005;365:1099–1104. 15781105 10.1016/S0140-6736(05)71146-6

[9] Coupe N, Cotterill S, Peters S. Tailoring lifestyle interventions to low socio-economic populations: a qualitative study. BMC Public Health 2018;18:967. 30075716 10.1186/s12889-018-5877-8 PMC6076398

[10] Moroshko I, Brennan L, O'Brien P. Predictors of dropout in weight loss interventions: a systematic review of the literature. Obes Rev 2011;12:912–934. 21815990 10.1111/j.1467-789X.2011.00915.x

[11] Armstrong K, Ravenell KL, McMurphy S, Putt M. Racial/ethnic differences in physician distrust in the United States. Am J Public Health 2007;97:1283–1289. 17538069 10.2105/AJPH.2005.080762 PMC1913079

[12] Paasche-Orlow MK, Wolf MS. The causal pathways linking health literacy to health outcomes. Am J Health Behav 2007;31:19–26. 10.5555/ajhb.2007.31.supp.S19 17931132

[13] Faber J.S., Al-Dhahir I, Kraal J.J., Breeman L.D., Reijnders T, Evers A.W.M., et al 37th Annual Conference of the European Health Psychology Society, Bremen, European Health Psychology Society. 2023.

[14] Fell J, Dale V, Doherty P. Does the timing of cardiac rehabilitation impact fitness outcomes? An observational analysis. Open Heart 2016;3:e000369. 26870390 10.1136/openhrt-2015-000369 PMC4746523

[15] Keessen P, van Duijvenbode IC, Latour CH, Kraaijenhagen RA, Janssen VR, Jørstad HT, et al Design of a remote coaching program to bridge the gap from hospital discharge to cardiac rehabilitation: intervention mapping study. JMIR Cardio 2022;6:e34974. 35612879 10.2196/34974 PMC9178457

[16] Sunamura M, Ter Hoeve N, Geleijnse ML, Steenaard RV, van den Berg-Emons HJG, Boersma H, et al Cardiac rehabilitation in patients who underwent primary percutaneous coronary intervention for acute myocardial infarction: determinants of programme participation and completion. Neth Heart J 2017;25:618–628. 28917025 10.1007/s12471-017-1039-3 PMC5653538

[17] Barnason S, Zimmerman L, Nieveen J, Schulz P, Young L. Patient recovery and transitions after hospitalization for acute cardiac events: an integrative review. J Cardiovasc Nurs 2012;27:175–191. 22210146 10.1097/JCN.0b013e318239f5f5

[18] Mai Ba H, Son Y-J, Lee K, Kim B-H. Transitional care interventions for patients with heart failure: an integrative review. Int J Environ Res Public Health 2020;17:2925. 32340346 10.3390/ijerph17082925 PMC7215305

[19] Neubeck L, Freedman SB, Clark AM, Briffa T, Bauman A, Redfern J. Participating in cardiac rehabilitation: a systematic review and meta-synthesis of qualitative data. Eur J Prev Cardiol 2012;19:494–503. 22779092 10.1177/1741826711409326

[20] Lie I, Bunch EH, Smeby NA, Arnesen H, Hamilton G. Patients’ experiences with symptoms and needs in the early rehabilitation phase after coronary artery bypass grafting. Eur J Cardiovasc Nurs 2012;11:14–24. 21030311 10.1016/j.ejcnurse.2010.09.004

[21] Timmins F, Kaliszer M. Information needs of myocardial infarction patients. Eur J Cardiovasc Nurs 2003;2:57–65. 14622649 10.1016/S1474-5151(02)00089-0

[22] Coleman EA, Parry C, Chalmers S, Min SJ. The care transitions intervention: results of a randomized controlled trial. Arch Intern Med 2006;166:1822–1828. 17000937 10.1001/archinte.166.17.1822

[23] Liao EN, Chehab LZ, Neville K, Liao J, Patel D, Sammann A. Using a human-centered, mixed methods approach to understand the patient waiting experience and its impact on medically underserved populations. BMC Health Serv Res 2022;22:1388. 36419056 10.1186/s12913-022-08792-8 PMC9682738

[24] Rao A, Zecchin R, Newton PJ, Phillips JL, DiGiacomo M, Denniss AR, et al The prevalence and impact of depression and anxiety in cardiac rehabilitation: a longitudinal cohort study. Eur J Prev Cardiol 2020;27:478–489. 31597473 10.1177/2047487319871716

[25] Clark AM, King-Shier KM, Thompson DR, Spaling MA, Duncan AS, Stone JA, et al A qualitative systematic review of influences on attendance at cardiac rehabilitation programs after referral. Am Heart J 2012;164:835–845.e2. 23194483 10.1016/j.ahj.2012.08.020

[26] Su JJ, Yu DS-F. Effects of a nurse-led eHealth cardiac rehabilitation programme on health outcomes of patients with coronary heart disease: a randomised controlled trial. Int J Nurs Stud 2021;122:104040. 34333211 10.1016/j.ijnurstu.2021.104040

[27] Goryakin Y, Thiebaut SP, Cortaredona S, Lerouge MA, Cecchini M, Feigl AB, et al Assessing the future medical cost burden for the European health systems under alternative exposure-to-risks scenarios. PLoS One 2020;15:e0238565. 32915826 10.1371/journal.pone.0238565 PMC7485835

[28] Moro Visconti R, Morea D. Healthcare digitalization and pay-for-performance incentives in smart hospital project financing. Int J Environ Res Public Health 2020;17:2318. 32235517 10.3390/ijerph17072318 PMC7177756

[29] Manteghinejad A, Javanmard SH. Challenges and opportunities of digital health in a post-COVID19 world. J Res Med Sci 2021;26:11. 34084190 10.4103/jrms.JRMS_1255_20 PMC8103966

[30] Scherrenberg M, Falter M, Dendale P. Cost-effectiveness of cardiac telerehabilitation in coronary artery disease and heart failure patients: systematic review of randomized controlled trials. Eur Heart J Digit Health 2020;1:20–29. 37056294 10.1093/ehjdh/ztaa005 PMC10087016

[31] Frederix I, Solmi F, Piepoli MF, Dendale P. Cardiac telerehabilitation: a novel cost-efficient care delivery strategy that can induce long-term health benefits. Eur J Prev Cardiol 2017;24:1708–1717. 28925749 10.1177/2047487317732274

[32] Kraal JJ, Van Den Akker-Van Marle ME, Abu-Hanna A, Stut W, Peek N, Kemps HM. Clinical and cost-effectiveness of home-based cardiac rehabilitation compared to conventional, centre-based cardiac rehabilitation: results of the FIT@Home study. Eur J Prev Cardiol 2017;24:1260–1273. 28534417 10.1177/2047487317710803 PMC5518918

[33] Reiners F, Sturm J, Bouw LJW, Wouters EJM. Sociodemographic factors influencing the use of eHealth in people with chronic diseases. Int J Environ Res Public Health 2019;16:645. 30795623 10.3390/ijerph16040645 PMC6406337

[34] Arsenijevic J, Tummers L, Bosma N. Adherence to electronic health tools among vulnerable groups: systematic literature review and meta-analysis. J Med Internet Res 2020;22:e11613. 32027311 10.2196/11613 PMC7055852

[35] Kerkhoff AD, Farrand E, Marquez C, Cattamanchi A, Handley MA. Addressing health disparities through implementation science-a need to integrate an equity lens from the outset. Implement Sci 2022;17:13. 35101088 10.1186/s13012-022-01189-5 PMC8802460

[36] Faber JS, Al-Dhahir I, Kraal JJ, Breeman LD, van den Berg-Emons RJG, Reijnders T, et al Guide development for eHealth interventions targeting people with a low socioeconomic position: participatory design approach. J Med Internet Res 2023;25:e48461. 38048148 10.2196/48461 PMC10728791

[37] Al-Dhahir I, Breeman LD, Faber JS, Reijnders T, van den Berg-Emons HJG., van der Vaart R, et al An overview of facilitators and barriers in the development of eHealth interventions for people of low socioeconomic position: a Delphi study. Int J Med Inform 2023;177:105160. 37549501 10.1016/j.ijmedinf.2023.105160

[38] Faber JS, Al-Dhahir I, Reijnders T, Chavannes NH, Evers AWM, Kraal JJ, et al Attitudes toward health, healthcare, and eHealth of people with a low socioeconomic status: a community-based participatory approach. Front Digit Health 2021;3:690182. 34713165 10.3389/fdgth.2021.690182 PMC8521920

[39] Vogelaar CP . Brief van de minister voor Wonen, Wijken en Integratie: Aanpak wijken (Vergaderjaar 2006–2007, 30 995, nr. 3). Tweede Kamer der Staten-Generaal. Sdu Uitgevers. 2007.

[40] Lund AM . Measuring usability with the USE questionnaire. STC Usability SIG Newsletter 2001;8:3–6.

[41] Bonevski B, Randell M, Paul C, Chapman K, Twyman L, Bryant J, et al Reaching the hard-to-reach: a systematic review of strategies for improving health and medical research with socially disadvantaged groups. BMC Med Res Methodol 2014;14:42. 24669751 10.1186/1471-2288-14-42 PMC3974746

[42] Chervinsky AB, Ommaya AK, deJonge M, Spector J, Schwab K, Salazar AM. Motivation for Traumatic Brain Injury Rehabilitation Questionnaire (MOT-Q): reliability, factor analysis, and relationship to MMPI-2 variables. Arch Clin Neuropsychol 1998;13:433–446. 14590608

[43] Williams AM, Lester L, Bulsara C, Petterson A, Bennett K, Allen E, et al Patient Evaluation of Emotional Comfort Experienced (PEECE): developing and testing a measurement instrument. BMJ Open 2017;7:e012999. 10.1136/bmjopen-2016-012999 PMC5278251 28122833

[44] Devilly GJ, Borkovec TD. Psychometric properties of the credibility/expectancy questionnaire. J Behav Ther Exp Psychiatry 2000;31:73–86. 11132119 10.1016/s0005-7916(00)00012-4

[45] Michie S, Richardson M, Johnston M, Abraham C, Francis J, Hardeman W, et al The behavior change technique taxonomy (v1) of 93 hierarchically clustered techniques: building an international consensus for the reporting of behavior change interventions. Ann Behav Med 2013;46:81–95. 23512568 10.1007/s12160-013-9486-6

[46] Oinas-Kukkonen H, Harjumaa M. Persuasive systems design: key issues, process model, and system features. Commun Assoc Inf Syst 2009;24:485–500.

[47] Asbjornsen RA, Wentzel J, Smedsrod ML, Hjelmesæth J, Clark MM, Solberg Nes L, et al Identifying persuasive design principles and behavior change techniques supporting end user values and needs in eHealth interventions for long-term weight loss maintenance: qualitative study. J Med Internet Res 2020;22:e22598. 33252347 10.2196/22598 PMC7735908

[48] Su JJ, Yu DSF. Effectiveness of eHealth cardiac rehabilitation on health outcomes of coronary heart disease patients: a randomized controlled trial protocol. BMC Cardiovasc Disord 2019;19:274. 31783800 10.1186/s12872-019-1262-5 PMC6884828

[49] Braun V, Clarke V. Using thematic analysis in psychology. Qual Res Psychol 2006;3:77–101.

[50] Crielaard L, Nicolaou M, Sawyer A, Quax R, Stronks K. Understanding the impact of exposure to adverse socioeconomic conditions on chronic stress from a complexity science perspective. BMC Med 2021;19:242. 34635083 10.1186/s12916-021-02106-1 PMC8507143

[51] Chou Y-k. The octalysis framework for gamification & behavioral design 2015. URL: https://yukaichou. com/gamification-examples/octalysis-complete-gamification-framework/ [accessed 2019-11-03].

[52] Yao R, Zhang W, Evans R, Cao G, Rui T, Shen L. Inequities in health care services caused by the adoption of digital health technologies: scoping review. J Med Internet Res 2022;24:e34144. 35311682 10.2196/34144 PMC8981004

[53] Choi NG, Dinitto DM. The digital divide among low-income homebound older adults: internet use patterns, eHealth literacy, and attitudes toward computer/internet use. J Med Internet Res 2013;15:e93. 23639979 10.2196/jmir.2645 PMC3650931

[54] Estacio EV, Whittle R, Protheroe J. The digital divide: examining socio-demographic factors associated with health literacy, access and use of internet to seek health information. J Health Psychol 2019;24:1668–1675. 28810415 10.1177/1359105317695429

[55] Kontos E, Blake KD, Chou WY, Prestin A. Predictors of eHealth usage: insights on the digital divide from the Health Information National Trends Survey 2012. J Med Internet Res 2014;16:e172. 25048379 10.2196/jmir.3117 PMC4129114

[56] Venkatesh V, Morris MG, Davis GB, Davis FD. User acceptance of information technology: toward a unified view. MIS Quart 2003;27:425–478.

[57] Brouwers RWM, Houben VJG, Kraal JJ, Spee RF, Kemps HMC. Predictors of cardiac rehabilitation referral, enrolment and completion after acute myocardial infarction: an exploratory study. Neth Heart J 2021;29:151–157. 33030659 10.1007/s12471-020-01492-0 PMC7904980

[58] Tenbult-Van Limpt N, Van Asten I, Brouwers R, Spee RF, Brini A, Kemps HMC. Information needs and information seeking behavior in patients receiving cardiac rehabilitation. Eur J Prev Cardiol 2022;29:zwac056.250.

[59] Yates BC, Vazquez Hernandez ML, Rowland SA, Bainter DE, Schulz P, Hanson CK. A qualitative study of experiences of participants in cardiac rehabilitation. J Cardiopulm Rehabil Prev 2018;38:E6–E9. 29485528 10.1097/HCR.0000000000000317 PMC6023746

[60] Doets EL, de Hoogh IM, Holthuysen N, Wopereis S, Verain MCD, van den Puttelaar J, et al Beneficial effect of personalized lifestyle advice compared to generic advice on wellbeing among Dutch seniors—an explorative study. Physiol Behav 2019;210:112642. 31394106 10.1016/j.physbeh.2019.112642

[61] Kaufmann C, Müller T, Hefti A, Boes S. Does personalized information improve health plan choices when individuals are distracted? J Econ BehavOrgan 2018;149:197–214.

[62] Tong HL, Quiroz JC, Kocaballi AB, Fat SCM, Dao KP, Gehringer H, et al Personalized mobile technologies for lifestyle behavior change: a systematic review, meta-analysis, and meta-regression. Prev Med 2021;148:106532. 33774008 10.1016/j.ypmed.2021.106532

[63] van den Brekel-Dijkstra K, Rengers AH, Niessen MA, de Wit NJ, Kraaijenhagen RA. Personalized prevention approach with use of a web-based cardiovascular risk assessment with tailored lifestyle follow-up in primary care practice—a pilot study. Eur J Prev Cardiol 2016;23:544–551. 26080811 10.1177/2047487315591441

[64] Aharon KB, Gershfeld-Litvin A, Amir O, Nabutovsky I, Klempfner R. Improving cardiac rehabilitation patient adherence via personalized interventions. PLoS One 2022;17:e0273815. 36037232 10.1371/journal.pone.0273815 PMC9423647

[65] Vosbergen S, Mulder-Wiggers JMR, Lacroix JP, Kemps HMC, Kraaijenhagen RA, Jaspers MWM, et al Using personas to tailor educational messages to the preferences of coronary heart disease patients. J Biomed Inform 2015;53:100–112. 25239261 10.1016/j.jbi.2014.09.004

[66] Skivington K, Matthews L, Simpson SA, Craig P, Baird J, Blazeby JM, et al A new framework for developing and evaluating complex interventions: update of Medical Research Council guidance. BMJ 2021;374:n2061. 34593508 10.1136/bmj.n2061 PMC8482308

[67] den Uijl I, Ter Hoeve N, Sunamura M, Stam HJ, Boersma E, Lenzen MJ, et al Cardiac rehabilitation designed for patients with obesity: OPTICARE XL RCT results on health-related quality of life and psychosocial well-being. Disabil Rehabil 2023;45:1046–1055. 35311438 10.1080/09638288.2022.2050428

[68] ter Hoeve N, Sunamura M, Stam HJ, Boersma E, Geleijnse ML, van Domburg RT, et al Effects of two behavioral cardiac rehabilitation interventions on physical activity: a randomized controlled trial. Int J Cardiol 2018;255:221–228. 29425564 10.1016/j.ijcard.2017.12.015

[69] Moon MD . Triangulation: a method to increase validity, reliability, and legitimation in clinical research. J Emerg Nurs 2019;45:103–105. 30616761 10.1016/j.jen.2018.11.004

