# Supplementary material for: An eHealth intervention for patients with a low socioeconomic position during their waiting period preceding cardiac rehabilitation: a randomized feasibility study
Source: Eur Heart J Digit Health. 2024 Nov 14;6(1):115–25. doi: 10.1093/ehjdh/ztae084 (PMC11750199; doi:10.1093/ehjdh/ztae084)
Supplement: ztae084_Supplementary_Data [file ztae084_supplementary_data.zip › Supplementary Appendix 1.pdf]

Supplementary Appendix 1- Questionnaires

Certainty and guidance

The following questions are about how confident and supported you feel after discharge from the hospital. For each statement, indicate to what extent you agree or disagree. Do this by circling the answer that most applies to your personal situation. We want to know what you personally think and not what you think the doctor or researcher wants to hear.

	Entirely Disagree	Disagree	Neutral	Agree	Entirely agree
I feel confident about my current situation.	○	○	○	○	○
I dare to carry out daily activities without being afraid that something will happen to me.	○	○	○	○	○
Rehabilitation will help me to become the way I was before the heart incident.	○	○	○	○	○
I know what to expect during cardiac	○	○	○	○	○
rehabilitation. In the past few days, I have felt	○	○	○	○	○
calm. I know well what is going on with me.	○	○	○	○	○
I know what I can do myself to improve my health.	○	○	○	○	○

Acceptance

How did you experience the app?

Please indicate below how you generally experienced using the app.

	Entirely Disagree	Disagree	Neutral	Agree	Entirely agree
The app is easy to use.	◯	◯	◯	◯	◯
Using the app required little effort from me.	◯	◯	◯	◯	◯
I could successfully use it every time.	◯	◯	◯	◯	◯
The app is useful.	◯	◯	◯	◯	◯
The app aligns with my needs.	◯	◯	◯	◯	◯
The app does everything I expected it to do.	◯	◯	◯	◯	◯
It is enjoyable to use the app.	◯	◯	◯	◯	◯
I would recommend the app to another participant.	◯	◯	◯	◯	◯
The app works the way I would like it to work.	◯	◯	◯	◯	◯
