# Supplementary material for: An eHealth intervention for patients with a low socioeconomic position during their waiting period preceding cardiac rehabilitation: a randomized feasibility study
Source: Eur Heart J Digit Health. 2024 Nov 14;6(1):115–25. doi: 10.1093/ehjdh/ztae084 (PMC11750199; doi:10.1093/ehjdh/ztae084)
Supplement: ztae084_Supplementary_Data [file ztae084_supplementary_data.zip › Supplementary Appendix 2.pdf]

Supplementary Appendix 2: Interview Guide

Feasibility
Adherence
At what moments did you typically use the app? Why?
Were there times when you didn't feel the need to use the app?

Acceptance
How did you find using the app?
What was your experience like?
What contributed to that?
How did you feel about having something during the waiting period? Or would you have preferred not to have it?
Was there anything else you felt was missing?
What would have helped you better cope with the waiting period?
What did you find difficult to use or understand in the app?

Elements
Which parts of the app did you find pleasant?
Receiving and reading messages every day
Little text -> watching videos and listening to voice messages
Achieving goals in between filling the meter and earning new travel bags
Appearance of the app
Language use + way of being addressed
Reminders Content
Stories from peers
Practical tips

Effect
If you had to summarize, where do you think the app helped you the most during the waiting period?
