# Supplementary material for: An eHealth intervention for patients with a low socioeconomic position during their waiting period preceding cardiac rehabilitation: a randomized feasibility study
Source: Eur Heart J Digit Health. 2024 Nov 14;6(1):115–25. doi: 10.1093/ehjdh/ztae084 (PMC11750199; doi:10.1093/ehjdh/ztae084)
Supplement: ztae084_Supplementary_Data [file ztae084_supplementary_data.zip › Supplementary Appendix 3.pdf]

Supplementary Appendix 3: Qualitative themes

Theme	F	Participants N (%)**	Description	Quote
**Intervention adherence**				
Fit in daily routine	24	13 (72)	Intervention use fitted in daily schedules and became a routine	*"We used to sit in the morning for coffee. Yeah, we would sit down for a while, and I received them, and then I had my phone in my hand. Well, I went through it, I even turned it on so that the lady could listen along and that way. Yeah, it's also at a fixed time. You have to be careful not to let it sit for a whole week and then review it after a week. Because that will not work, I think. If you throw everything together, it is just a matter of sifting through it and fulfilling a duty."*
Curiosity	17	11 (61)	Curiosity about new messages	*"I was curious about it every day. I also opened it every day. I went through the entire program. I was, well, actually, looking forward to see what news they had to say today. Yeah, it was actually more curiosity."*
**Usability and satisfaction**				
Easy to use	23	18 (100)	Finds the intervention easy to use	*"Yes, I find it quite easy, actually. You take the train, and then you grab that other thing, the suitcase, and then you just move it back and forth a bit. And that went well. That all went smoothly."*
Appreciates playfulness	13	13 (72)	Appreciates the playful design of the interface	*"Well, you know, I found it enjoyable. More enjoyable than just a boring list or something, you know. Yeah, it's funny that they thought of it like, oh yeah, let's pretend it's a journey. With your stories in a suitcase, very amusing. You're on a journey to your rehabilitation."*
Appreciates peer stories	9	7 (39)	Appreciates the integration of peer stories functionality	*"Yes, and stories from others, right? People naturally relate to that. Because there's always a story like, oh, I've experienced that too. And then I don't have to worry about it, so to speak."*
Reward system unclear	7	7 (39)	Did not understand the travel bag upgrade system	*"Those numbers that were next to it, at one point, it was on fourteen, then it went back to seven or something, just to give you an idea. Oh, why is that then?"*
**Content and Language**				
Lack of depth and detail	26	10 (56)	Finds information too superficial and needs an additional layer of depth.	*"Look, we're talking about the dietitian. And the dietitian gave a very brief explanation of what she does. Very brief, I can't remember exactly how it was explained. But she didn't really delve into the topic. For example, what can you tell about your sugar or salt levels being too high? What are the consequences of that? Could you get paralysis? Could you have a heart attack? So, the information was lacking, in my opinion. More substance is needed."*
Clear communication	21	16 (89)	Found the information easy to comprehend	*"At least, for me, it was easy to follow, not too difficult. No, let's say, it wasn't overly technical language. Also not overly simplified, but simply clear in terms of what was said and expected, yes."*
Need for personalized information	17	11 (61)	Needs information that is personalized to individual disease situations and preferences	*"All those social workers and such... For me, I think, it's not interesting. I only do it to become physically well. That's my goal. I don't think I have any other issues. And then, yes... I think the app is limited in that aspect."*
Need for personalized advice	11	6 (33)	Needs advice that is personalized to individual disease situations and preferences	*"See, one piece of advice was to go for a walk. For example, walk to the supermarket. Well, I can walk a bit further as well."*
**Impact and Relevance**				

Rehabilitation roadmap	22	12 (67)	Felt the intervention provided a clear understanding of what can be expected during CR	*"You know, when all those people introduced themselves and told stories about different participants. Yeah, that was nice because you get an idea in advance of what to expect when you start the rehabilitation. So, that was quite pleasant."*
Certainty during transition	12	5 (28)	Felt the intervention brought a sense of certainty during the waiting period	*"Well, in terms of reducing uncertainties, the app did help me because if you didn't have that app, you would fall into a void between being discharged from the hospital and starting rehabilitation. So, in that sense, the app was able to provide assistance in filling that void at some point."*
Health Status Understanding	10	7 (39)	Felt the intervention Provided comprehensive information about the patient's current situation.	*"I received a lot of information that I wouldn't normally get. If you haven't had a heart attack, you don't even think about all the information you've received. So, for me, it was a kind of recognition. And, actually, it was very good. So, as I said, it made me wiser."*
Managing emotions	5	4 (22)	Felt the intervention Helped to improve emotional wellbeing	*"Yeah, as I mentioned. The first time for me, last Monday, entering that room and getting back into motion. That was scary. And that had already been told in a story. Something I had heard. And it did sound familiar. In the story, I also heard that once you take that first step, it gets better. And that was absolutely true."*
Pre-rehabilitation guidance	5	4 (22)	Felt the intervention provided guidance in activities that can already be done while waiting for rehabilitation to begin.	*"Well, yeah, at least that I have to pay attention, that I have to balance my diet a bit, and also that I have to start exercising, and yes, everything helped."*
Movement Confidence	1	1 (6)	Felt the intervention helped to feel less fear of medical incidents when moving or undertaking activities.	*"I thought, well, I'll go along on the bike for once. And, yeah, we happened to have a headwind, and then, well, you start to feel a bit uneasy on that bike. And, yeah, then you also think back to those videos where it was said, for example, one starts too fast and the other starts too slow, thinking they can't do it anymore, and so on. And then you think about... that. And it actually helps with cycling because the next day I got back on the bike. And, yeah, that's how you overcome some of the fear you have about, well, doing the activities you need to do."*
Hope	0	0 (0)	Felt the intervention provided a clear understanding of a positive outlook in the future health journey	
**Integration in care journey**				
Transition missing	11	7 (39)	Transistion from end-of-use of the intervention towards rehabilitation is missing	*"Yeah, what I missed, of course, is that at some point, it stops. And then you only see the conductor, I believe. And he says, well, it's going well like this. And then I think to myself, well, what's going so well, that the train isn't running, but you don't get advice. And that's actually when the therapy is in sight, then the train keeps going. You miss the point where the advice stops. And in a few days, you get therapy. And then we continue with the physical part."*
**Technical issues**				
No new messages issue	7	5 (28)	Mentioning of a technical issue in which participant	*"At some point, nothing was coming through to me, so I thought, is this all there is? I didn't find it very helpful, what exactly is the intention? But then it turned out okay after all."*

			didn't receive new messages	

*F = Frequency
** of the 18 interviewed participants
